# Influence of AlN(0001) Surface Reconstructions on the Wettability of an Al/AlN System: A First-Principle Study

**DOI:** 10.3390/ma11050775

**Published:** 2018-05-11

**Authors:** Junhua Cao, Yang Liu, Xiao-Shan Ning

**Affiliations:** State Key Laboratory of New Ceramics and Fine Processing, School of Materials Science and Engineering, Tsinghua University, Beijing 100086, China; caojunhuamenghuan@163.com (J.C.); stillwaters.liu@163.com (Y.L.)

**Keywords:** surface reconstruction, first-principle, Al/AlN interface, work of adhesion, contact angle

## Abstract

A successful application of a hot dip coating process that coats aluminum (Al) on aluminum nitride (AlN) ceramics, revealed that Al had a perfect wettability to the ceramics under specific circumstances, which was different from previous reports. In order to elucidate the mechanism that controlled the supernormal wetting phenomenon during the dip coating, a first-principle calculation of an Al(111)/AlN(0001) interface, based on the density functional theory (DFT), was employed. The wettability of the Al melt on the AlN(0001) surface, as well as the effect that the surface reconstruction of AlN and the oxygen adsorption had on Al for the adhesion and the wettability of the Al/AlN system, were studied. The results revealed that a LCM (laterally contracted monolayer) reconstruction could improve the adhesion and wettability of the system. Oxygen adsorption on the free surface of Al decreased the contact angle, because the adsorption reduced of the surface tension of Al. A prefect wetting was obtained only after some of the oxygen atoms adsorbed on the free surface of Al. The supernormal wetting phenomenon came from the surface reconstruction of the AlN and the adsorption of oxygen atoms on the Al melt surface.

## 1. Introduction

Aluminum nitride (AlN) ceramics possess excellent thermal and dielectric properties, where they have been widely utilized as packaging substrates of a high power semiconductor after surface metallization [1]. Aluminum (Al) is suitable for the metallization, because it has a high electrical conductivity and it is soft, meaning that it does not exert excessive thermal stress on the AlN ceramic substrates after they bond. Al has the potential to improve the thermal shock tolerance property, which is one of the most important properties of the substrate. However, Al is easily oxidized in air, and the oxide film hinders its ability to bond with ceramics. The wettability of the Al melt on the ceramics is also poor, making coating Al onto ceramics extremely difficult [2]. To solve this problem, a low-pressure plasma spraying method [3], molecular beam epitaxy (MBE) [4,5], or magnetron sputtering methods [6] have been used to coat the Al onto the ceramics. Our research group has recently proposed a more convenient and simple hot dip coating method in order to coat Al onto these ceramics [7]. This method allows a 5 μm thick Al film to be coated on the surface of AlN ceramics [8,9,10]. 

A perfect wetting is essential for the hot dip coating process, because the liquid that is attached on the solid substrate must spread over the substrate to form a film. Numerous experiment results have revealed that a uniform film is formed on the solid only when the equilibrium contact angle of a liquid on a solid is less than 20°, or when the receding contact angle reaches 0° [11]. However, the equilibrium contact angle of the Al melt on the AlN ceramics, which has been measured by a lot of research groups, is higher than 75° under 1073 K [12,13,14], which is also a typical temperature in the hot dip coating experiments. Therefore, a supernormal wetting phenomenon actually occurs in the hot dip coating of Al on the AlN ceramics. Furthermore, such a supernormal wetting phenomenon was also observed in the fabrication of composite materials. Nautiyal et al. [15] used a molten route method to fabricate the Al/BN (boron nitride) composite materials in the air at 973 K. They observed that a layer of the AlN had formed on the surface of the BN, as a result of the interface reaction, which led to a tight combination with the Al. The perfect wetting of Al on AlN is key to the successful fabrication of the Al/BN composite materials. 

We experimentally confirmed that the oxygen content, the temperature, and the soak time influenced the soundness of the dip-coated Al film [8]. The experimental condition of the hot dip coating was compared with the contact angle measurement to find the following differences:

(1) The AlN ceramics are soaked in an Al melt during a hot dip coating experiment, which means that they are in an Al-rich and N-poor environment. This differs from the contact angle measurement, where the AlN ceramics are typically maintained in a high vacuum environment.
(2) There are dozens of parts per million (ppm) of oxygen in the atmosphere of the dip coting equipment that were used in experiment, which is much higher than the residual oxygen that is found in the contact angle measurement’s high vacuum environment.

**Figure 1:**
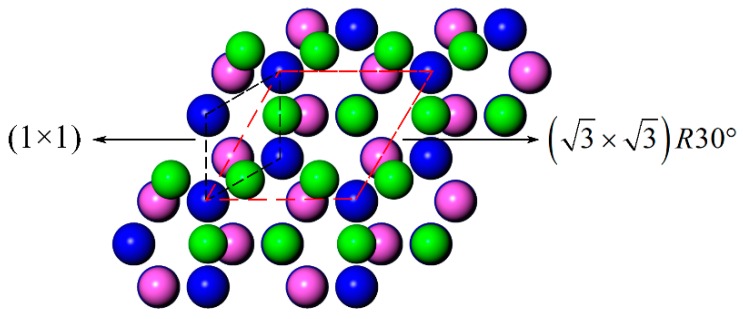
Top views of the LCM aluminum nitrate (AlN)(0001)/aluminum (Al)(111) interface models. The red balls represent the Al atoms of the AlN(0001) slab, the blue balls represent N atoms, and the green balls represent the Al atoms of the laterally contracted Al monolayer.

Previous reports have demonstrated that an Al-terminated AlN(0001) surface would reconstruct in an Al-rich and N-poor environment [16,17]. Lee et al. [16] has observed several types of reconstruction RHEED (reflection high-energy electron diffraction) patterns from a 700–800 °C MBE AlN(0001) surface, including 2 × 2, 1 × 3, 2 × 6, and so on. They studied the structures and the stability of several AlN(0001) surface reconstructions using first-principle calculations. They found that a 2 × 2 structure of N adatom at the H_3_ site is stable in N-rich conditions, a 2 × 2 structure of Al adatom at the T_4_ site becomes stable as the Al chemical potential increases, and a $\left(\sqrt{3}\;\times \;\sqrt{3}\right)R30{}^{\circ}$ structure of the Al laterally contracted monolayer (LCM, see Figure 1) is more stable in Al-rich conditions. Similar results have been obtained by other researchers [17,18,19]. In the hot dip coating experiment, an LCM reconstruction will also form on the AlN(0001) surface, because of the similar Al-rich environment. 

The surface reconstructions could influence the wettability of the Al on the AlN ceramics. Liu et al. [20] studied the wettability of the Al melt on the Al_2_O_3_(0001) surface by a first-principle calculation, and found that the Al on the Al_2_O_3_(0001)$\;\left(\sqrt{31}\;\times \;\sqrt{31}\right)R\;\pm \;9{}^{\circ}$ reconstruction surface had a better wettability than that on an Al_2_O_3_(0001) 1 × 1 surface. In this work, the AlN(0001)/Al(111) interface was chosen as a model for the first-principle calculations. The impact that the surface reconstruction and experiment condition had on the wettability of the system was detailed in the calculation, in order to illuminate the theory of supernormal wetting in the hot dip coating of Al on AlN ceramics.

## 2. Method

### 2.1. Calculation Method

The calculations in this work were based on the Density Functional Theory (DFT) and used the *Vienna* Ab initio Simulation Package [21]. A plane-wave basis set was used for the expansion of the single-particle Kohn–Sham wave functions. The generalized gradient approximation of the Perdew–Burke–Ernzerhof (GGA-PBE) [22] was employed to so as to approximate the exchange-correlation energy. The interaction between the ions and electrons was described by the projector-augmented wave (PAW) method [23], which has been shown to be in good agreement with other pseudo potentials and exchange-correlation functions [16,18,19].

Our calculation method was validated by the calculation of the lattice constants of thee bulk Al and AlN. The calculated lattice constants of Al were a = b = c = 4.049 Å, which were in good agreement with the experimental value of 4.03 Å [24], and a calculation value of 4.039 Å, which was previously obtained [25]. The calculation results were a = b = 3.112 Å, c/a = 1.602 for the lattice constants of AlN, which was in good agreement with the experimental value (a = b = 3.11 Å, c/a = 1.602 [26]) and the previous calculation results [19]. 

**Figure 2:**
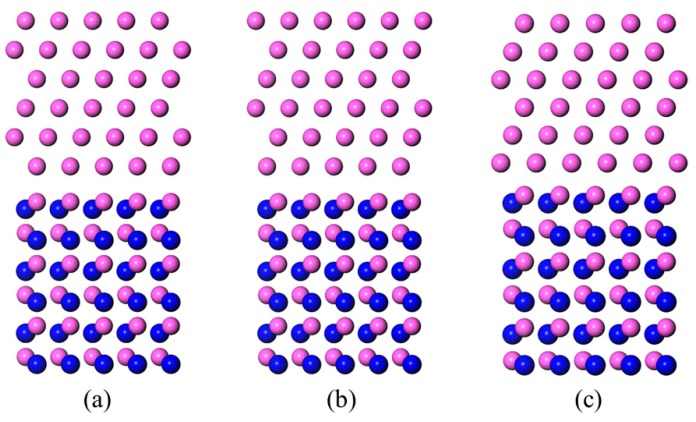
Side views of the Al-terminated AlN(0001)/Al(111) interface models before structure optimization. The red balls represent the Al atoms and the blue balls represent the N atoms. (**a**) Al-T_A_; (**b**) Al-T_B_; and (**c**) Al-T_C_.

**Figure 3:**
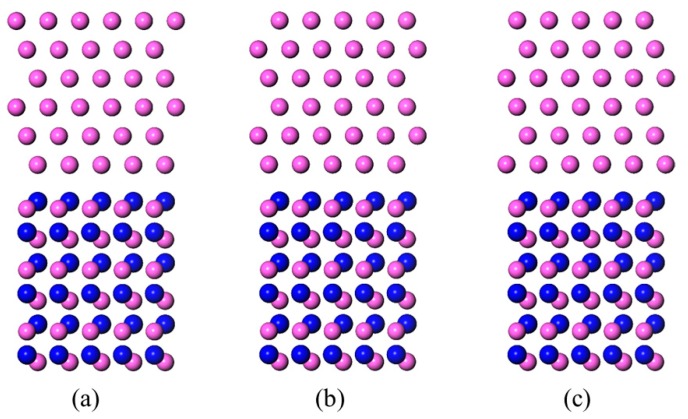
Side views of the N-terminated AlN(0001)/Al(111) interface models before structure optimization. The red balls represent the Al atoms and the blue balls represent the N atoms. (**a**) N-T_A_; (**b**) N-T_B_; and (**c**) N-T_C_.

**Figure 4:**
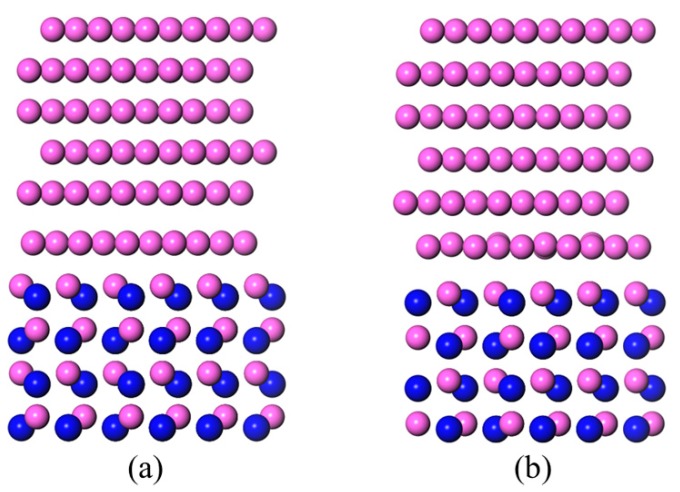
Side views of the LCM AlN(0001)/Al(111) interface models. The red balls represent the Al atoms and blue balls represent N atoms. (**a**) Before structure optimization and (**b**) after structure optimization.

Montesa et al. [27] observed that the Al-Si alloy/AlN interface by HRTEM (high resolution transmission electron microscope) and found an orientation relationship of Al(111)$\langle 1\bar{1}0\rangle$||AlN(0001)$\langle 11\bar{2}0\rangle$. The Al/AlN interface models were based on these findings. Six Al layers of Al(111), and three Al–N bilayers of AlN(0001) were contained in each interface model. Both of the Al terminated and N terminated conditions of the AlN slabs were taken into consideration. Three different models were established, which were dependent on the matchup between the Al(111) slab and the AlN(0001) slab. The outmost Al atom layer of the Al(111) slab was sited on top of the surface atom (type A), subsurface atom (type B), and a hollow position (type C) of the AlN(0001) slab, see Figure 2 and Figure 3. In the LCM reconstructed interface model (Figure 4a), the AlN(0001) slab contained four Al–N bilayers and the Al(111) slab contained five Al layers. A laterally contracted monolayer of Al atoms was added on top of the AlN slab. The slight mismatch (about 7%) between the Al(111) slab and the AlN(0001) slab was deemed to be absorbed through the distortion of the soft Al. A 10 Å thick vacuum slab was added on the top and bottom of the models, in order to prevent interactions between the slabs and their mirror images. A 9 × 9 × 1 k-points mesh was chosen for all of the models, so as to ensure both accuracy and efficiency.

### 2.2. Work of Adhesion and Contact Angle

The contact angle (*θ*) is usually used to describe the wettability, which is defined by the Young’s Equation, as follows:

(1) $$\sigma_{sv}\;=\;\sigma_{sl}\;+\;\sigma_{lv}\cdot \cos\left(\theta \right)$$

where $\sigma_{sv}$, $\sigma_{sl}$, and $\sigma_{lv}$ are the surface or interface energy of the solid-vapor, the solid-liquid, and the liquid-vapor interfaces. The work of the adhesion (*W_ad_*) is also important, which is defined as the work that is required to separate an interface into two free surfaces without considering the elastic deformation and diffusion [28]:

(2) $$W_{ad}\;=\;\sigma_{sv}\;+\;\sigma_{lv}\;-\sigma_{sl}$$

The *W_ad_* is linked with *θ* by Young–Dupré’s Equation, as follows:

(3) $$W_{ad}\;=\;\sigma_{lv}\cdot [1\;+\;\cos(\theta )]$$

Strictly, the contact angle, which is calculated by the equation, should be a receding one, in consideration of the definition of *W_ad_*. *W_ad_* can be calculated by the following equations:

(4) $$W_{ad}{\;=\;(E}_{slab\left(Al\right)}^{tot}{\;+\;E}_{slab\left(ALN\right)}^{tot}\;-{\;E}_{interface}^{tot})/A$$

where $E_{slab\left(Al\right)}^{tot}$ and $E_{slab\left(ALN\right)}^{tot}$ are the total energy of the Al slab and the AlN slab, and $E_{interface}^{tot}$ is the total energy of the interface model. *A* is the area of the interface.

## 3. Results and Discussion

### 3.1. Al-Terminated AlN(0001)/Al(111) Interface Models

**Figure 5:**
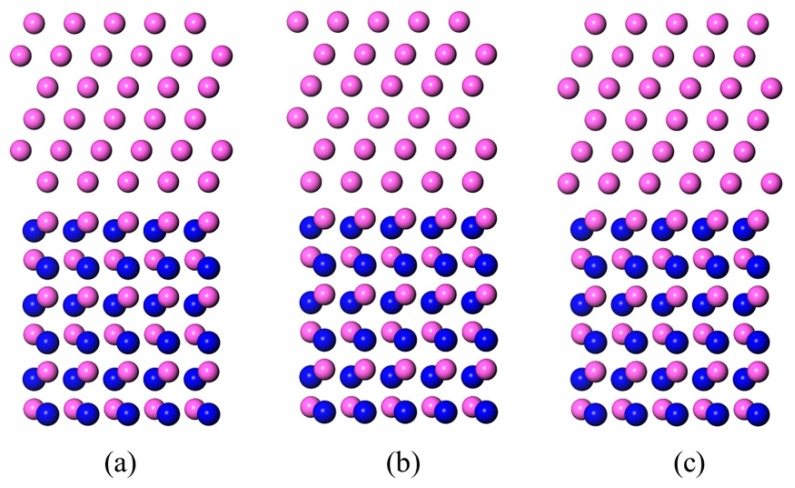
Side views of the Al-terminated AlN(0001)/Al(111) interface models after the structure optimization. The red balls represent the Al atoms and the blue balls represent the N atoms. (**a**) Al-T_A_; (**b**) Al-T_B_; and (**c**) Al-T_C_.

The optimized structures of the Al-terminated AlN(0001)/Al(111) interface models are shown in Figure 5. Compared with the original models (Figure 2), fewer position changes near the interface were found throughout the three models, indicating a favorable stability. 

The calculated work of the adhesion (*W_ad_*) and contact angle (*θ*) throughout the three interface models is listed in Table 1. There were minor differences between the values of the surface tension of the Al melt at 1073 K by different researchers [29,30,31], so the calculated contact angle was a bit scattered. The temperature, 1073 K, was approximately the same as in the dip coating. The results demonstrated that the contact angle values of the three models were higher than 83°, which agreed well with the results of many sessile drop experiments [12,13,14]. In the work of Kumamoto et al. [32], a 2.46 J/m^2^ *W_ad_* was obtained in an Al/AlN interface model, which was similar to the Al-T_A_ model that was presented in this work. However, such a *W_ad_* led to a 0° contact angle, which apparently disagreed with the experiments results [12,13,14]. The smaller k-points mesh that was used in their calculation, 3 × 3 × 1, might have been responsible for the deviation. 

### 3.2. N-Terminated AlN(0001)/Al(111) Interface Models

**Figure 6:**
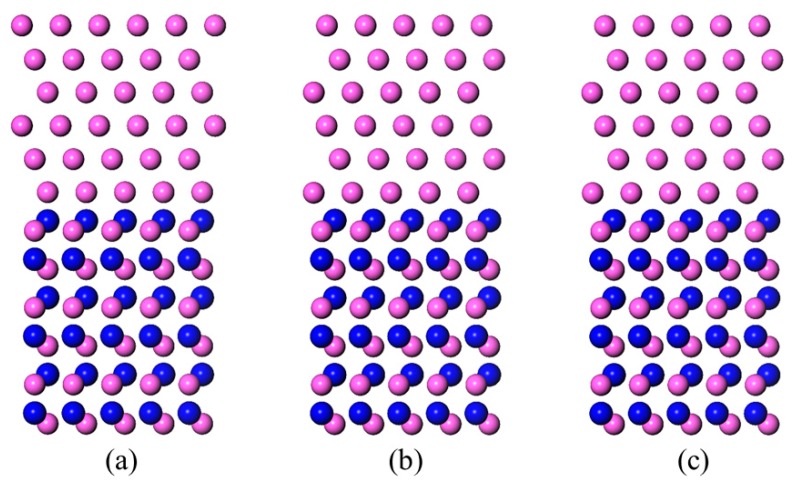
Side views of the N-terminated AlN(0001)/Al(111) interface models after the structure optimization. The red balls represent the Al atoms and the blue balls represent the N atoms. (**a**) N-T_A_; (**b**) N-T_B_; and (**c**) N-T_C._

The optimized structures of the Al-terminated AlN(0001)/Al(111) interface models are shown in Figure 6. Compared with the original models (Figure 3), the distance between the Al(111) slab and the AlN(0001) slab was shortened throughout the three models. The outmost aluminum atoms of the Al(111) slab in the N-T_B_ model was close to the hollow position, which was similar to the N-T_C_ model.

The calculated work of the adhesion (*W_ad_*) and contact angle (*θ*) at 1073 K throughout the three interface models were calculated and are displayed in Table 2. The results of the N-T_B_ and N-T_C_ models were similar. The *W_ad_* was 0.82 J/m^2^ and the contact angles were about 106°~107°, which dissatisfied the requirement for the hot dip coating experiments. The calculated results of the N-T_A_ model were different. The *W_ad_* was up to 2.40 J/m^2^, which was significantly larger than the other models. As a result, the contact angle of the Al melt on the N-T_A_ surface reached 0°, revealing a good wettability. 

**Figure 7:**
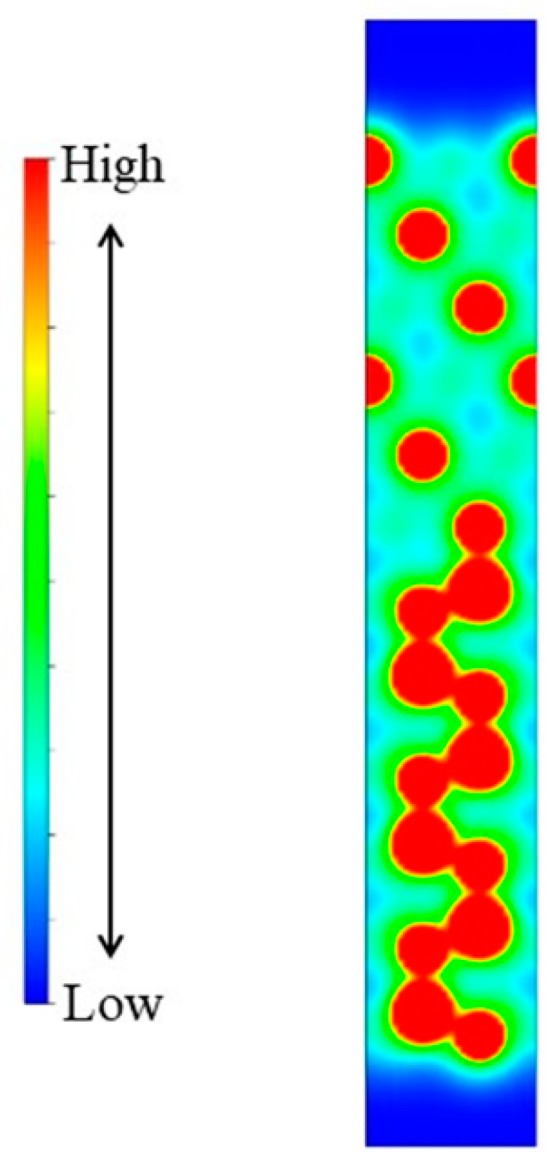
Electron density schematic diagram of the optimized N-T_A_ model (110) surface.

The distance between the bottommost Al atoms of the Al(111) slab and the topmost N atoms of the AlN(0001) slab was 1.92 Å, after the structure optimization. This was close to the distance of the nearest Al and N layers of 1.90 Å. According to the electron density schematic diagram of the N-T_A_ model (110) surface (Figure 7), the electron distribution near the interface was similar to the AlN(0001) slab. These two results illustrated that the stable chemical bonds that were formed between the terminated N atoms of the AlN(0001) and the Al atoms of the Al(111) slab in the interface, which had subsequently increased the *W_ad_*. This was beyond the scope of the work of the adhesion, however a reconstruction surfaced on the N-terminated AlN(0001) surface. During the hot dip coating experiments, the N-T_A_ structure was more stable and more likely to appear, which suggested that the N-terminal AlN(0001) surface chemically reacted with the Al melt so as to bond with a layer of Al atoms. In an Al-rich environment, the N-terminated AlN(0001) surface tended to bond the Al atoms and transformed into an Al-terminated surface.

### 3.3. LCM Reconstructed AlN(0001)/Al(111)

After the structure optimization (Figure 4b), a few position changes were found, and the laterally contracted Al monolayer showed some motion along a vertical axis. The calculated work of the adhesion (*W_ad_*) and contact angle (*θ*) at 1073 K of the LCM interface models were calculated and listed in Table 3. The *W_ad_* showed an evident increase when compared with the original Al-terminated AlN(0001)/Al(111) interface models, which demonstrated that the combination of the AlN(0001) slab and the Al(111) slab was tighter. The calculated contact angle of the Al melt on the LCM AlN(0001) surface had decreased to 35°~41°. The LCM reconstruction improved the wettability, but the contact angle was still far from the 0° that the hot dip coating experiment required.

### 3.4. Influence of Oxygen Adsorption on Free Surface of Al Melt

**Figure 8:**
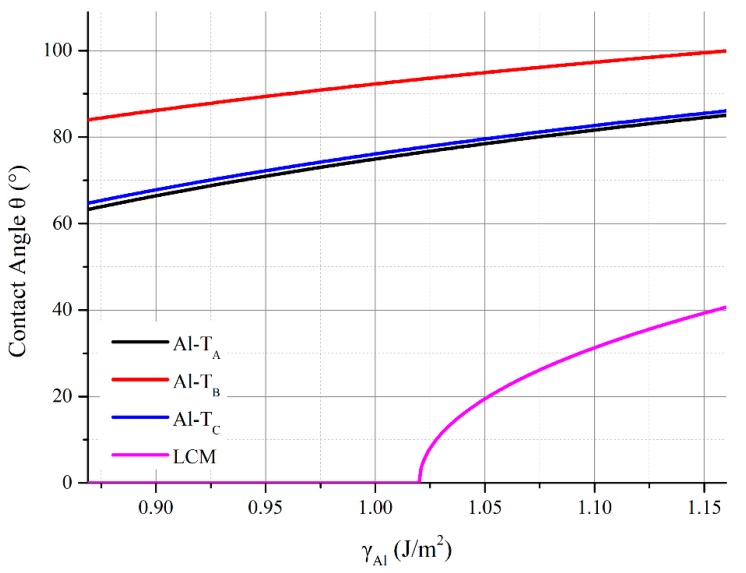
Diagram of the calculated contact angle of the molten Al on the Al-terminated and the LCM AlN(0001) surface at 1073 K, as a function of surface tension of Al.

During the hot dip coating experiment, there were dozens of ppm of oxygen in the atmosphere of the dip coating equipment. Garcia-Cordovilla et al. [31] had observed a decrease in the surface tension of the Al melt as the oxygen (O) atoms were adsorbed onto the surface of the melt. The previous experiment measured a surface tension value of 0.869 J/m^2^ at 1073 K as a monolayer of the O atoms that were adsorbed. According to Young–Dupré’s Equation, the contact angle would have declined with the decrease in the surface tension of the Al melt. We calculated the contact angle as the surface tension changing from 0.869 J/m^2^ to 1.160 J/m^2^, and results were shown in Figure 8. Throughout the three original Al-terminated AlN(0001) surfaces, the contact angle of the Al melt on the Al-T_A_, Al-T_B_, and Al-T_C_ surfaces decreased to 63°, 84°, and 65° when the *γ_Al_* had decreased to 0.869 J/m^2^ . The contact angle of the Al melt on the LCM surface decreased to 0° when the *γ_Al_* had decreased to 1.02 J/m^2^. A perfect wetting was obtained after a partial adsorption of the O atoms. The supernormal wetting phenomenon that was observed in the hot dip coating likely came from the surface reconstruction of the AlN and the adsorption of the O atoms on the free surface of the Al film, which was attached on the AlN.

## 4. Conclusions

In order to characterize the supernormal wetting phenomenon that occurred in the hot dip coating of the Al on the AlN ceramics, the wettability of the Al melt on the AlN(0001) surface was studied by a first-principle study. A surface reconstruction of the Al-terminated AlN(0001) surface and the oxygen adsorption were taken into consideration. The conclusions were as follows:

(1) The *W*_ad_ of the Al-terminated AlN(0001)/Al(111) was within the limits of 0.96 J/m^2^~1.26 J/m^2^, where the calculated contact angle of the Al melt on the Al-terminated AlN(0001) surface was beyond 83°, which agreed well with the sessile drop experiments.
(2) The *W*_ad_ of the N-T_B_ and N-T_C_ types N-terminated AlN(0001)/Al(111) interface was 0.82 J/m^2^, which resulted in a 106°~107° calculated contact angle of the Al melt on the N-T_B_ and N-T_C_ surfaces, at 1073 K. The *W*_ad_ of the N-T_A_ type of the AlN(0001)/Al(111) interface was 2.40 J/m^2^, which resulted in a 0° calculated contact angle of the Al melt on the N-T_A_ surface at 1073 K. This was caused by the chemical bonds that formed between the terminated N atoms on the AlN(0001) surface and the Al atoms of the Al melt. The results demonstrated that, in an Al-rich environment, the N-terminated AlN surface tended to bond the Al atoms and reconstructed, which resulted in an Al-terminated surface structure.
(3) The LCM reconstruction improved the Al and AlN bonding in the Al-terminated AlN(0001) surface. The *W*_ad_ of the LCM AlN(0001)/Al(111) interface was 2.04 J/m^2^, which was higher than the 0.96 J/m^2^~1.26 J/m^2^ of the dis-reconstructed interface. The wettability of the Al on the AlN was improved and the contact angle of the Al on the AlN(0001) surface decreased from 83°~100° to 35°~41°.
(4) The adsorption of the oxygen atoms on the free surface of the Al melt further improved the wettability of the Al. The contact angle between the Al melt and the non-reconstructed Al-terminated AlN(0001) surface decreased from 83°~100° to 63°~84°. When one monolayer of oxygen atoms was adsorbed on the surface of the Al melt, the surface tension decreased from 1.122~1.160 J/m^2^ to 0.869 J/m^2^. The partial adsorption of the oxygen atoms decreased the contact angle to 0° when the surface tension decreased to 1.02 J/m^2^ within the LCM reconstruction.
(5) The supernormal wetting phenomenon in the dip coating of the Al on AlN ceramics originated from the surface reconstruction of the AlN ceramics under an Al-excess condition when they were immersed in molten Al. A quick adsorption occurred for the oxygen atoms on the free surface of liquid Al, which were attached on the AlN ceramics, as they withdrew from the Al melt. A thorough immersion and a proper amount of oxygen in the atmosphere were key for the hot dip coating.

## Figures and Tables

**Table 1:** Calculated work of the adhesion (*W_ad_*) and contact angles (*θ*) of the aluminum (Al)-terminated aluminum nitride (AlN)(0001)/Al(111) interface models at 1073 K.

Interface Model	Work of Adhesion *W*_ad_ (J/m^2^)	Contact Angle *θ* (°)		
		*γ_Al_* = 1.143 J/m^2^ [29]	*γ_Al_* = 1.160 J/m^2^ [30]	*γ_Al_* = 1.122 J/m^2^ [31]
Al-T_A_	1.26	84	85	83
Al-T_B_	0.96	99	100	98
Al-T_C_	1.24	85	86	84

**Table 2:** Calculated work of adhesion (*W_ad_*) and contact angles (*θ*) of the N-terminated AlN (0001)/Al (111) interface models at 1073 K.

Interface Model	Work of Adhesion *W*_ad_ (J/m^2^)	Contact Angle *θ* (°)		
		*γ_Al_* = 1.143 J/m^2^ [29]	*γ_Al_* = 1.160 J/m^2^ [30]	*γ_Al_* = 1.122 J/m^2^ [31]
N-T_A_	2.40	0	0	0
N-T_B_	0.82	107	107	106
N-T_C_	0.82	107	107	106

**Table 3:** Calculated work of the adhesion (*W_ad_*) and contact angles (*θ*) of the LCM AlN(0001)/Al(111) interface models at 1073 K.

Interface Model	Work of Adhesion *W*_ad_ (J/m^2^)	Contact Angle *θ* (°)		
		*γ_Al_* = 1.143 J/m^2^ [29]	*γ_Al_* = 1.160 J/m^2^ [30]	*γ_Al_* = 1.122 J/m^2^ [31]
LCM	2.04	38	41	35
